# Predominant transmission of KPC-2 carbapenemase in Germany by a unique IncN plasmid variant harboring a novel non-transposable element (NTE*_KPC_*-Y)

**DOI:** 10.1128/spectrum.02564-23

**Published:** 2023-12-12

**Authors:** Yancheng Yao, Linda Falgenhauer, Yalda Rezazadeh, Jane Falgenhauer, the IncN Study Group, Anja M. Hauri, Can Imirzalioglu, Trinad Chakraborty

**Affiliations:** 1 Hessisches Landesprüfungs- und Untersuchungsamt im Gesundheitswesen - HLPUG, Dillenburg, Germany; 2 Justus Liebig University Giessen, Giessen, Germany; 3 German National Reference Centre for Multidrug-Resistant Gram-negative Bacteria, Ruhr-University Bochum, Bochum, Germany; 4 University of Bonn, Bonn, Germany; 5 Leibniz-Institute DSMZ-German Collection of Microorganisms and Cell Cultures, Braunschweig, Germany; 1 Institute of Medical Microbiology, Justus Liebig University Giessen, Giessen, Germany; 2 German Center for Infection Research (DZIF), Partner Site Giessen-Marburg-Langen, Giessen, Germany; 3 Institute for Hygiene and Environmental Medicine, Justus Liebig University Giessen, Giessen, Germany; University of California, San Diego, La Jolla, California, USA

**Keywords:** KPC-2, IncN, plasmid-mediated transmission, non-Tn4401-element, carbapenem resistance, *Enterobacterales*

## Abstract

Carbapenemase-producing *Enterobacterales* (CPE) pose a major public health threat. Despite active infection prevention efforts, the incidence of KPC-2 carbapenemase-producing *Enterobacterales* (KPC2-CPE) continues to increase worldwide. In this study, we performed genome sequencing of 135 KPC2-CPE isolates obtained from multiple sources (clinical, hospital environments, and surface water) in Germany between 2013 and 2019 and analyzed them for epidemiological clues regarding transmission. For 92% (124/135) of all isolates, which comprised 14 different species such as *Klebsiella, Escherichia, Citrobacter, Enterobacter, Raoultella,* and *Serratia*, KPC-2 was present on an IncN[pMLST15] plasmid. All plasmids carried a novel non-Tn*4401*-element harboring an *aac (3)-IId-bla*_TEM-1B_-*bla*_KPC-2_–cassette (designated NTE*_KPC_*-Y) that was co-transferred with an adjacent region carrying 12 further antibiotic resistance genes. Identical plasmids were also detected in KPC2-CPE isolates from environmental samples. These plasmids were remarkably stable and were maintained in individual patients colonized with KPC-2 CPEs over a long-term period (>1 year). Thus, a predominant broad host range signature IncN[pMLST15] plasmid mediates transmission of both KPC-2 and associated multiple antimicrobial drug resistance genes in Germany. These data underline the need for in-depth characterization of plasmid carriers of CPE in surveillance and outbreak studies as well as in microbiomes from patients and the environment to identify hidden transmission reservoirs. This information will be essential for the development and implementation of effective infection control and prevention measures to disrupt dissemination of KPC2- CPEs in healthcare and associated environmental settings.

**IMPORTANCE:**

Current infection control protocols assume that the spread of KPC-2 carbapenemase-producing *Enterobacterales (*KPC2-CPE) by detected carriers to other in-house patients is through clonal transmission and can be restricted by implementing containment measures. We examined the presence of the *bla*_KPC-2_ gene in different genera and species of *Enterobacterales* isolated from humans at different hospitals and surface waters between 2013 and 2019 in Germany. We found that a single IncN[pMLST15] plasmid carrying the *bla*_KPC-2_ gene on a novel non-Tn*4401*-element (NTE_KPC_-Y), flanked by an adjacent region encoding 12 other antibiotic resistance genes, was uniquely present in multiple species of KPC2-CPE isolates. These findings demonstrate the selective impact of specific IncN plasmids as major drivers of carbapenemase dissemination and suggest “plasmid-based endemicity” for KPC2-CPE. Studies on the dynamics of plasmid-based KPC2-CPE transmission and its presence in persistent reservoirs need to be urgently considered to implement effective surveillance and prevention measures in healthcare institutions.

## INTRODUCTION

Infections caused by carbapenemase-producing *Enterobacterales* (CPE) are increasing, and the lack of safe and efficacious treatment options presents a challenge for healthcare systems and patient safety around the globe (1). *Klebsiella pneumoniae* carbapenemase (KPC) is one of the leading causes of carbapenem resistance in nosocomial infections and CPE outbreaks worldwide (2 – 4). Current data suggest that these outbreaks are usually locally limited in nature and tend to be restricted to medical facilities, often subsiding following the implementation of infection prevention and control interventions (5). Nevertheless, the incidence of KPC-2-dependent infections worldwide remains high and continues to increase (6, 7).

Dissemination of *bla*_KPC-2_ involves horizontal transfer of *bla*_KPC-2_-encoding genetic elements and plasmids (8). A retrospective cohort study in Singapore suggested that plasmid-mediated transmission accounted for ~45% of all CPE carriers (9). Plasmids were predominantly of the incompatibility groups (Inc) N and FII, followed by R, A/C2, P, L/M, Q1, FIB, U, FIA, X3, Q2, and N3 (10, 11). Conjugal transfer of carbapenemase-encoding plasmids occurs in different species that have adapted to various ecological niches (12). Inanimate environments and particularly the aqueous environment represent reservoirs for facilitating plasmid-based transmission of carbapenemase genes in hospital settings (13). Dissemination of antibiotic resistance genes (ARGs) via plasmids represents a significant but poorly understood challenge in community health and hospital infection control.

The *bla*_KPC-2_ gene is often found embedded within a ~ 10-kb transposon Tn*4401* (14 – 17), which was, for example, present in 35.5% (72/192) of all KPC-2 plasmids in the study by Brandt et al. (10). This composite transposon is predominant in epidemiologically successful *K. pneumoniae* ST258 complex clones (15, 18). However, reports of the presence of *bla*_KPC-2_ within non-Tn*4401*-elements (NTE*_KPC_*) are now frequent and increasing (5, 17, 19). Unlike the transposable Tn*4401* element, NTE*_KPC_* elements lack the flanking IS*Kpn7* and the Tn*3* family transposase and resolvase. By using additional genes adjacent to *bla*_KPC-2_ for classification, three NTE*_KPC_* groups, viz., NTE*_KPC_*-I, NTE*_KPC_*-II and NTE*_KPC_*-III, have been described (17). Members of the NTE*_KPC_*-II group (with its three subgroups IIa, IIb, and IIc) carry a truncated *bla*_TEM_ sequence upstream of *bla*_KPC-2_ flanked by a partial IS*Kpn6* element, downstream. These NTE*_KPC_*-II subgroups are often associated with different plasmid types (17, 20). Here, we report for the first time on the detection of an NTE_KPC_ variant harboring both *bla*_KPC-2_ and a complete *bla*_TEM-1B_ gene on a unique group of IncN plasmids, which concomitantly also transfers multiple adjacent antibiotic resistance genes to generate pan-resistant CPEs.

## RESULTS

### Origins and genetic diversity of the KPC-2 carbapenemase-producing *Enterobacterales* (KPC2-CPE)

In a 3-year genome-based surveillance study between 2017 and 2019 of 61 hospitals in the German Federal State of Hessen, we obtained a total of 589 carbapenem-resistant Gram-negative bacteria isolates. Of these, 346 were carbapenemase-producing *Enterobacterales* (CPEs). The genotypes detected were OXA-48-like (40.5%), KPC- (31.6%), NDM- (17.8%), and VIM- (15.1%). Notably, carbapenemase genes were detected in 149 of 188 *K*. *pneumoniae* isolates and 86 of 105 *E. coli* isolates, as well as in 49 of 52 *Citrobacter,* 15 of 41 *Enterobacter,* four of four *Raoultella,* and 20 of 21 *Serratia* isolates (21).

**Fig 1:**
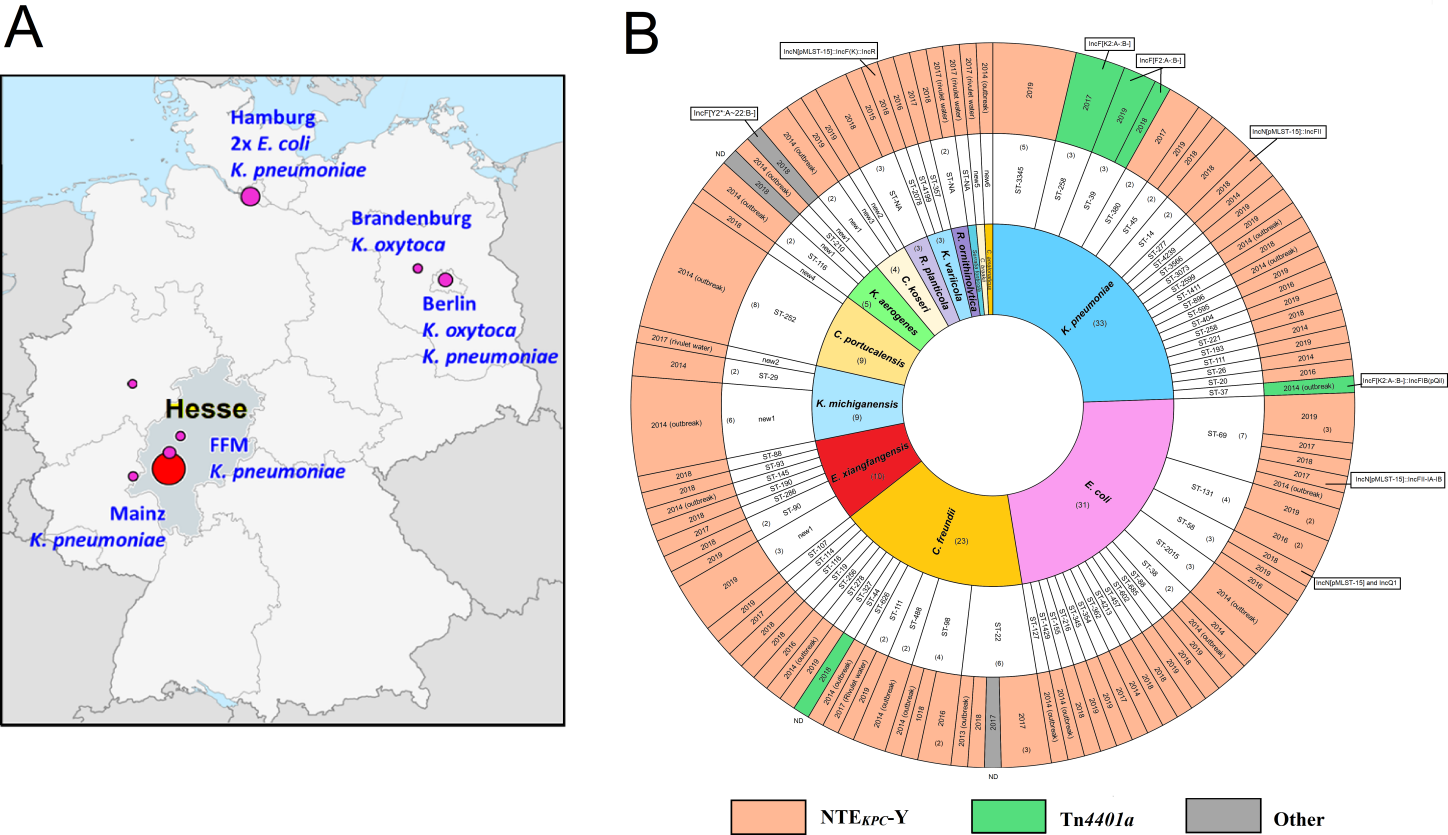
(**A**) Geographic location of *bla*_KPC-2_-encoding IncN[pMLST15] plasmids identified from the outbreak and various hospitals in Hesse as well as in the rivulet water in Germany. (The base map is reproduced from https://commons.wikimedia.org/wiki/File:Germany_location_map.svg#/media/File:Germany_location_map.svg, by NordNordWest, licensed under the Creative Commons Attribution-Share Alike 3.0 Unported license.) (**B**) Overview of the 135 KPC-2 carbapenemase-producing isolates with characteristics of the taxonomic and genome sequence types as well as the distribution of the genetic elements surrounding the gene *bla*_KPC-2_ (NTE_KPC_-Y and Tn*4401a*). All the NTE_KPC_-Y-harboring *bla*_KPC-2_–plasmids identified as Inc-type IncN[pMLST15] in single replicon (*n* = 120) or as co-integrates of multiple replicon types (*n* = 4). Most of the Tn*4401a* –containing *bla*_KPC-2_–plasmids belonged to IncF(*K*) or IncF(*II*) groups. The year of isolation of the respective isolates is depicted in the outermost ring.

KPC-2 carbapenemase-producing CPE (KPC2-CPE) represented ≈ 25% (*n* = 85) of all CPEs, and this percentage remained annually constant over the entire study period. For comparison, we included 37 KPC2-CPE isolates from a nosocomial outbreak from 2013/14 in Southern Hesse, Germany (22) and eight additional KPC2-CPE isolates obtained from patients in the German Federal States of Hesse, Hamburg, Berlin, and Brandenburg obtained between 2014 and 2015 (Fig. 1A). Five KPC2-CPEs collected from the surface water (2017) of a rivulet where the suspected index patient of an outbreak in the Rhine-Main region suffered a near-drowning incident were also included in the study (23).

KPC-2-producing isolates included those from rectal swabs (*n* = 73), urine samples (*n* = 12), respiratory samples (*n* = 12), bloodstream-infections (*n* = 2), and other sites (*n* = 21). Isolates from the hospital environment associated with the outbreak (*n* = 10) and rivulet water samples (*n* = 5) were included in the study. Detailed information of all KPC2-CPE isolates, including their genomic characteristics, plasmid Inc types, and antimicrobial resistance genes, is presented in Table S1.

Genomic taxonomy and multi-locus sequence typing (MLST) analysis revealed high taxonomic and genetic diversity among the 135 KPC2-CPE isolates. We detected 14 species comprising the genera *Klebsiella, Escherichia, Citrobacter, Enterobacter, Raoultella,* and *Serratia* that were distributed into 74 individual MLST types (Fig. 1B). The most commonly occurring species were *K. pneumoniae* (22 STs, *n* = 33), *E. coli* (16 STs, *n* = 31), *C. freundii* (10 STs, *n* = 23), and *E. xiangfangensis* (7 STs, *n* = 10).

### A predominant IncN[pMLST15] plasmid carrying a novel NTE_KPC_-Y cassette

For 124 isolates of KPC2-CPEs, the *bla*_KPC-2_ gene was located on IncN plasmids that were subtyped as pMLST15. Of these, 121 were single-replicon IncN[pMLST15] plasmids and three were cointegrates of this plasmid with IncF-, IncFII-, and IncQ1 plasmids (Table 1; Table S2). In eight of the remaining 11 KPC2-CPE isolates, the *bla*_KPC-2_ gene was located on IncF plasmids. Seven of them were associated with a Tn*4401a* element, whereas in three isolates, no definite assignment of the location on either the chromosome or plasmid was possible (Table S3). Within the surveillance period, IncN[pMLST15] was detected in 75 of 85 KPC2-CPE from 10 different species and associated with more than 46 MLST types. These isolates were obtained from 45 patients in 19 hospitals distributed across the State of Hesse.

**TABLE 1:** Contribution of the *bla*_KPC-2_ containing NTE_KPC_-Y-carrying IncN[pMLST15] plasmids based on antimicrobial resistance gene (ARG) profiles in Germany from 2013 to 2019 (*n* = 124)^*c*^ ^,^ ^*d*^

Study category and sampling period (year)	SurvCARE Hesse (2017–2019)	Outbreak in Hesse (2013–2014)	Nation-wide KPC-2 CPE (2014–2015)	Rivulet water screening (2017)
Sequenced KPC-2-carrying isolates	85	37	8	5
IncN[pMLST15] plasmids carrying NTE_KPC_-Y	75^*a*^	36	8	5
Percentages	88%	97%	100%	100%
Occurrence in species below: no. of isolates and (ST-Types):				
*C. amalonaticus*	-	1 (ND)	-	-
*C. braakii*	2 (ND)	-	-	1 (ND)
*C. freundii*	14 (9)	6 (5)	-	1 (1)
*C. koseri*	2 (ND)	2 (ND)	-	-
*C. portucalensis*	1 (1)	8 (1)	-	-
*E. coli*	21 (11)	*7* (5)	2 (2)	-
*E. cloacae*	3 (3)	-	-	-
*E. xiangfangensis*	6 (4)	1 (1)	-	-
*K. aerogenes*	-	3 (2)	-	-
*K. michiganensis*	-	6 (1)	2 (2)	1 (1)
*K. pneumoniae*	22 (14)	2 (2)	3 (3)	-
*K. variicola*	3 (3)	-	-	-
*R. ornithinolytica*	1 (ND)	-	-	1 (ND)
*R. planticola*		-	1 (ND)	-
*S. fonticola*	2 (ND)	-	-	1 (ND)
Sampling sources:				
No. of isolates from humans	75	24	8	0
No. of patients	50	19	8	0
No. of hospitals	19	3	8	-
No. of isolates from the environment	0	12	0	5
ARG profile^*b*^				
A	45	31	8	5
B	2	-	-	-
C	7	-	-	-
D	2	-	-	-
E	13	4	-	-
F	2	1	-	-
Various	4	-	-	-

^*a*^ Includes multi-replicon plasmids of IncN[pMLST15] co-integrated either with IncF[F36+31:A4:B10], IncF(K2:A-:B-]+IncR,, or IncF[K9:A-:B-].

^*b*^ The details of antibiotic resistance genes of ARG profiles are shown in Table 2.

^*c*^ SurvCARE Hesse: a regional genome-based CRE surveillance study (State of Hesse).

^*d*^ ND: not determined.

**TABLE 2:** ARG profiles of the *bla*_KPC-2_-bearing IncN[pMLST15] plasmids

ARG profile	Antibiotic resistance genes	Median size of single IncN replicon plasmid (Min–Max) in bps	Size of cointegrates (IncN plus IncF/IncR) in bps
A	*bla*_KPC-2_, *bla*_TEM-1B_, *bla*_OXA-1_, *strA, strB, aac(6´)-Ib-cr, aac (3)-IId, qnrB2, mph(A), catB3, aar-3, sul1 (1–2x), dfrA19* and *qacE∆−1*	78,021 (68,547–89,053)	195,038 or 216,551
B	*bla*_KPC-2_, *bla*_TEM-1B_, *bla*_OXA-1_, *aac(6´)-Ib-cr, aac (3)-IId, qnrB2, mph(A), catB3, aar-3, sul1 (1–2x), dfrA19,* and *qacE∆−1;*	73,409–83,074	
C	*bla*_KPC-2_, *bla*_TEM-1B_, *bla*_OXA-1_, *strA, strB, aac(6´)-Ib-cr, aac (3)-IId, catB3, aar-3, sul1 (1–2x), dfrA19,* and *qacE∆−1*	69,943 (64,969–71,469)	
D	*bla*_KPC-2_, *bla*_TEM-1B_, *bla*_OXA-1_, *aac(6´)-Ib-cr, aac (3)-IId, catB3, aar-3, sul1 (1–2x), dfrA19,* and *qacE∆−1*	67,754	164,049
E	*bla*_KPC-2_, *bla*_TEM-1B_, *strA, strB,* and *aac (3)-IId*	59,478 (58,446–70,547)	
F	*bla*_KPC-2_, *bla*_TEM-1B_, and *aac (3)-IId*	47,540	

### Overview of *bla*_KPC-2_-bearing plasmids characterized by long-read Sequencing

For detailed analysis of *bla*_KPC-2_-bearing plasmids, we subjected a subset of the KPC2-CPE (*n* = 34) to long-read sequencing. The isolates selected for sequencing broadly covered species and multi-locus sequence types together with the year of collection, i.e., between 2013 and 2019, and their KPC-2-encoding plasmids are given in Table 3. This plasmid data set was from 11 species that included eight *E. coli* of six STs (ST58, ST69, ST127, ST131, ST362, and ST2015), six *K*. *pneumoniae* (ST14, ST37, ST111, ST277, ST1411, and ST3345), six *C*. *freundii* (ST22, ST98, ST278, and ST327), three *K*. *variicola* of ST357, ST20178, and ST4199, as well as two- of *E. xiangfangensis*, *C. koseri*, *C. portucalensis* and one- each of *C. amalonaticus*, *K. aerogenes*, *R. ornithinolytica*, and *R. planticola*. Isolates were obtained in 2013 (1), 2014 (9), 2016 (1), 2017 (5), 2018 (12), and 2019 (6). Of the 34 long-read-sequenced KPC2-CPEs, 32 carried a unique IncN[pMLST15] plasmid replicon with the allele combination *repN*_7/*korA*_3/*traJ*_6, and the remaining two were IncF plasmids (Table 3). The plasmids with a single IncN[pMLST15] replicon were generally 78 kb in size, but plasmid variants with sizes ranging from 52 kb to 89 kb were also detected.

**TABLE 3:** Genomic characteristics of the *bla*_KPC-2_-bearing plasmids based on long-read sequencing

Plasmid name	Size (kb)	Inc type and pMLST	*bla*_KPC-2_-harboring genetic elements	ARG-profile	Species	Genome accession no.
pCF13141-KPC2	78	IncN[pMLST15]	NTE_KPC_-Y	A	*C. freundii*	VKMY01000050
pCF08698-KPC2	78	IncN[pMLST15]	NTE_KPC_-Y	A	*C. freundii*	VKMD01000077
pCP13069-KPC2	78	IncN[pMLST15]	NTE_KPC_-Y	A	*C. portucalensis*	VKMZ01000118
pCK13142-KPC2	78	IncN[pMLST15]	NTE_KPC_-Y	A	*C. koseri*	VKNB01000047
pKP37361-KPC2	78	IncN[pMLST15]	NTE_KPC_-Y	A	*K. pneumoniae*	CP104944
pEClo_Surv151-KPC2	78	IncN[pMLST15]	NTE_KPC_-Y	A	*E. xiangfangensis*	CP104949
pKV30046-KPC2	78	IncN[pMLST15]	NTE_KPC_-Y	A	*K. variicola*	CP104940
pCPsc18-2783-KPC2	78	IncN[pMLST15]	NTE_KPC_-Y	A	*C. portucalensis*	CP104956
pEC16155-KPC2	79	IncN[pMLST15]	NTE_KPC_-Y	A	*E. coli*	VKML01000077
pCA13304-KPC2	77	IncN[pMLST15]	NTE_KPC_-Y	A	*C. amalonaticus*	VKME01000146
pEXva18-1651-KPC2	79	IncN[pMLST15]	NTE_KPC_-Y	A	*E. xiangfangensis*	CP104948
pCK_Surv347-KPC2	79	IncN[pMLST15]	NTE_KPC_-Y	A	*C. koseri*	CP104957
pEC32446-KPC2	69	IncN[pMLST15]	NTE_KPC_-Y	A^*a*^	*E. coli*	CP104950
pEC14408-2-KPC2	88	IncN[pMLST15]	NTE_KPC_-Y	A	*E. coli*	LT599827
pCFur18-0060-KPC2	89	IncN[pMLST15]	NTE_KPC_-Y	A	*C. freundii*	CP104958
pCF37969-KPC2	89	IncN[pMLST15]	NTE_KPC_-Y	A	*C. freundii*	CP104960
pEC32009-KPC2	195	IncN[pMLST15]:: IncF[F36 +31:A4:B10]	NTE_KPC_-Y	A	*E. coli*	CP104951
pKV_Surv097-KPC2	217	IncN[pMLST15]:: IncFII(K2:A-:B-]::IncR	NTE_KPC_-Y	A	*K. variicola*	CP104941
pCF38954-KPC2	80	IncN[pMLST15]	NTE_KPC_-Y	D	*C. freundii*	CP104959
pCF_Surv457-KPC2	83	IncN[pMLST15]	NTE_KPC_-Y	B^*a*^	*C. freundii*	CP104961
pRP_Surv085-KPC2	73	IncN[pMLST15]	NTE_KPC_-Y	B	*R. planticola*	CP104937
pEC_Surv265-KPC2	69	IncN[pMLST15]	NTE_KPC_-Y	D	*E. coli*	CP104953
pKP38941-KPC2	69	IncN[pMLST15]	NTE_KPC_-Y	C	*K. pneumoniae*	CP104943
pKP41623-KPC2	164	IncN[pMLST15]::IncFII[K9:A-:B-]	NTE_KPC_-Y	D	*K. pneumoniae*	CP104942
pRO41724-KPC2	71	IncN[pMLST15]	NTE_KPC_-Y	E	*R. ornithinolytica*	CP104938
pKP_Surv398-KPC2	59	IncN[pMLST15]	NTE_KPC_-Y	E	*K. pneumoniae*	CP104946
pKP_Surv434-KPC2	59	IncN[pMLST15]	NTE_KPC_-Y	E	*K. pneumoniae*	CP104945
pKV33665-KPC2	59	IncN[pMLST15]	NTE_KPC_-Y	E	*K. variicola*	CP104939
pEC_Surv291-KPC2	59	IncN[pMLST15]	NTE_KPC_-Y	E	*E. coli*	CP104952
pCF12908-KPC2	59	IncN[pMLST15]	NTE_KPC_-Y	E	*C. freundii*	VKMA01000036
pEC_Surv190-KPC2-1	52	IncN[pMLST15]	NTE_KPC_-Y	E	*E. coli*	CP104954
pEC_Surv190-KPC2-2	23	IncQ1-like	NTE_KPC_-Y	E^*b*^	*E. coli*	CP104955
pEC11992-KPC2	48	IncN[pMLST15]∆	NTE_KPC_-Y	F	*E. coli*	VKMQ01000091
pKA36387-KPC2	166	IncF[Y2^*a*^:A ~ 22:B-]	Other^*c*^	Other^*d*^	*K. aerogenes*	CP104947
pKP11394-KPC2	241	IncF[K2:A-:B-]::IncFIB(*pQil*) :IncFIB(*pQil*)	Tn*4401a*	Other^*e*^	*K. pneumoniae*	VKNL01000120

^*a*^ these both plasmids carried only one copy of *sul1.*

^*b*^ pEC_Surv190-KPC2-2 harbored an E-like profile without *strA,*, but *sul2.*

^*c*^ pKA36387-KPC2 harbored neither a NTE_KPC_-Y nor Tn*4401.*

^*d*^ *bla*_KPC-2_ and *bla*_TEM-1_ are not adjacent to each other.

^*e*^ pKP11394-KPC2 harbored two Tn*4401a* with *bla*_KPC-2_.

**Fig 2:**
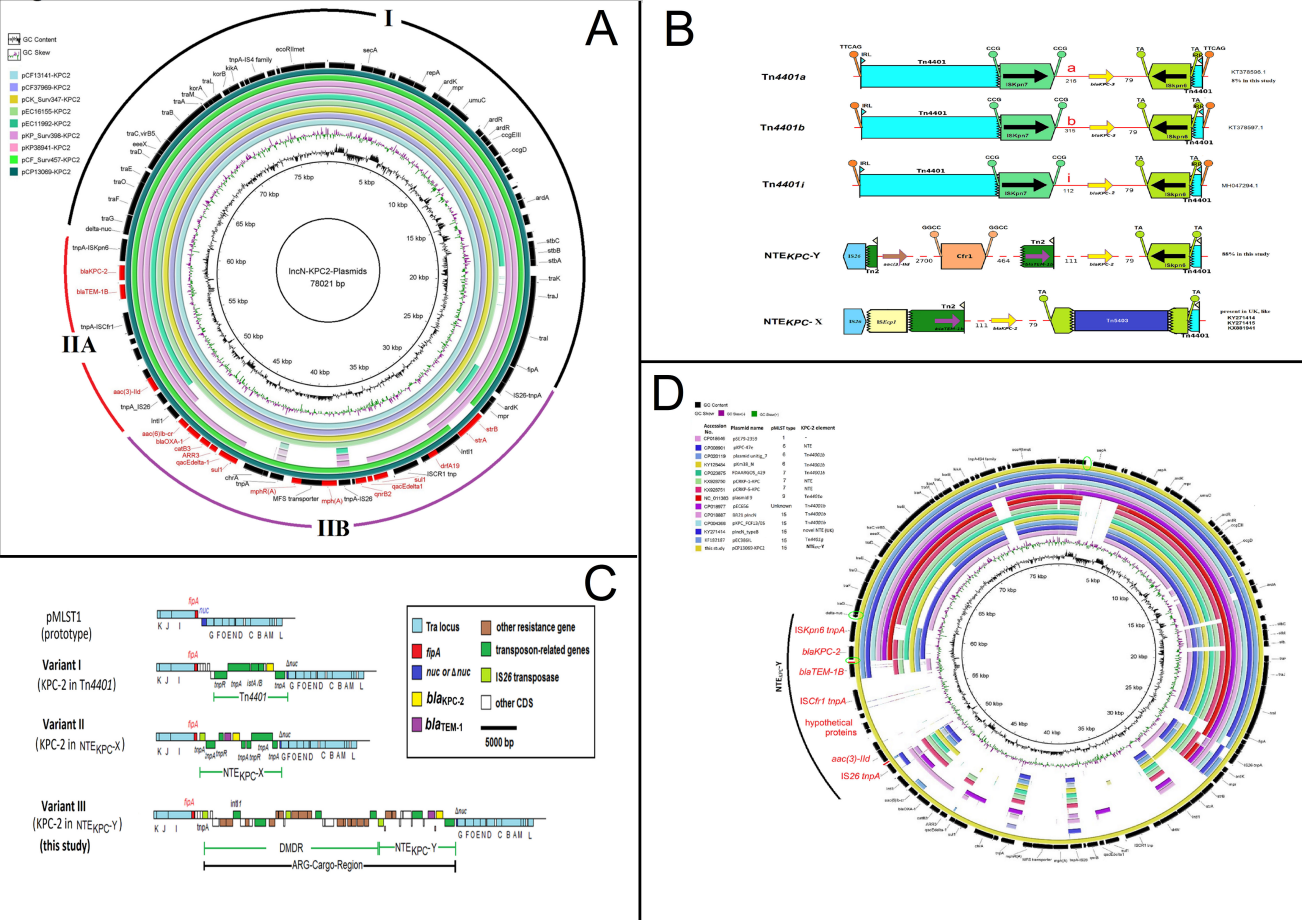
(**A**) Genetic map of the representative complete IncN[pMLST15] plasmids with the common length of 78,021 bp depicting the backbone (region I), the unique genetic environment NTE_KPC_-Y (region IIA), and the dynamic multi-drug resistance region (DMDR; Region IIB). Antimicrobial resistance genes (ARGs) are marked in red. The genetic environment of the NTE_KPC_-Y (region IIA), encoding *bla*_KPC-2_–*bla*_TEM-1B_ and *acc (3)IId*, was present in all plasmids; the DMDR (region IIB) differed in some plasmids, representing different antimicrobial resistance profiles. (**B**) Comparison of the non-Tn*4401*-KPC-element NTE_KPC_-Y (this study), NTE_KPC_-X (from United Kingdom), and Tn*4401* variants from the KPC-2-encoding IncN[pMLST15] plasmids. (**C**) Insertion sites of the ARG-bearing cargo-region of different IncN[pMLST15] variants. Variant I, *bla*_KPC-2_ located in Tn*4401* (CP004366, CP004367, CP018963, KX154765, KX276209, KX062091, KX397572, KF182187, and KF181264); variant II, *bla*_KPC-2_ located in and NTE_KPC_-X (KX88194, KY27414, and KY27415); and variant III, the *bla*_KPC-2_, was located in NTE_KPC_-Y (this study). (**D**) Comparison of different KPC-2 IncN pMLST types. The pCP13069-KPC2 contained a unique region, assigned as NTE_KPC_-Y. The green circles on the ring maps symbolize the areas for the specific PCR primers.

The composite structure of the IncN[pMLST15] plasmids consists of a 43-kb IncN-characteristic backbone (region I), which includes the replication region, a conjugation (*tra*) system, a stability operon, and an anti-restriction system, similar to that of pNL194, an IncN(pMLST18) plasmid (24). The *tra* genes are separated into three sections TRA-I, TRA-II, and TRA-III (24). The region between TRA-II and TRA-III (comprising *fipA* and *nuc*) is separated by the insertion of an acquired segment of ~35 kb (region II) into the gene *nuc*, and this region II can in turn be divided into two segments, region IIA and IIB. Region IIA (11 kb) comprises a section carrying three ARGs including *bla*_KPC-2_ on a non-Tn*4401*-element (described in detail as follows), which is commonly present in all IncN[pMLST15] plasmids regardless of size (Fig. 2A).

Region IIB (24 kb) is a mosaic structure that carries a complex array of 12 additional ARGs conferring resistance to nine antibiotic classes, including aminoglycosides (*strA*; *strB*), beta-lactams (*bla*_OXA-1_), fluoroquinolones (*aac(6´)-Ib-cr*, *qnrB2*), macrolides [*mph(A*)], phenicoles (*catB3*), rifampicin (ARR-3), sulfonamides (*sul1*, two copies), trimethoprim (*dfrA19*), and quaternary ammonium compounds (*qacE*Δ*_1*). These ARGs are associated with numerous insertion sequences (ISs) and transposons, for example, two IS*26*, IS*CR1*, IS*6100*, ΔTn5393, ΔTn402, and ΔTn*ch* and a class 1 integron (Fig. 2A). Region IIB is prone to deletions and can also harbor transposon insertions that account for variability in plasmid length, as, for example, with Tn*5075* (1327 bp) in pExva18651-KPC2 and Tn*5403* (2564 bp) in pEcSurv291-KPC2. In addition, an IS*903* flanked by direct repeat sequences (GCGCATGGC) and a truncated Tn*Pa38* (Tn*3* family transposon) were present in region I.

Plasmid transfer experiments with the donor strains CF08098 and CP13069 showed *bla*_KPC-2_ IncN[pMLST15] transconjugants with plasmids of different sizes (~78 kb and ~60 kb), indicating transfer of resistance to multiple antibiotic classes as well as an inherent instability of the ARGs structure in region II (Fig. S1).

### A novel non-Tn*4401*-KPC2-element (NTE_KPC_-Y)

**Fig 3:**
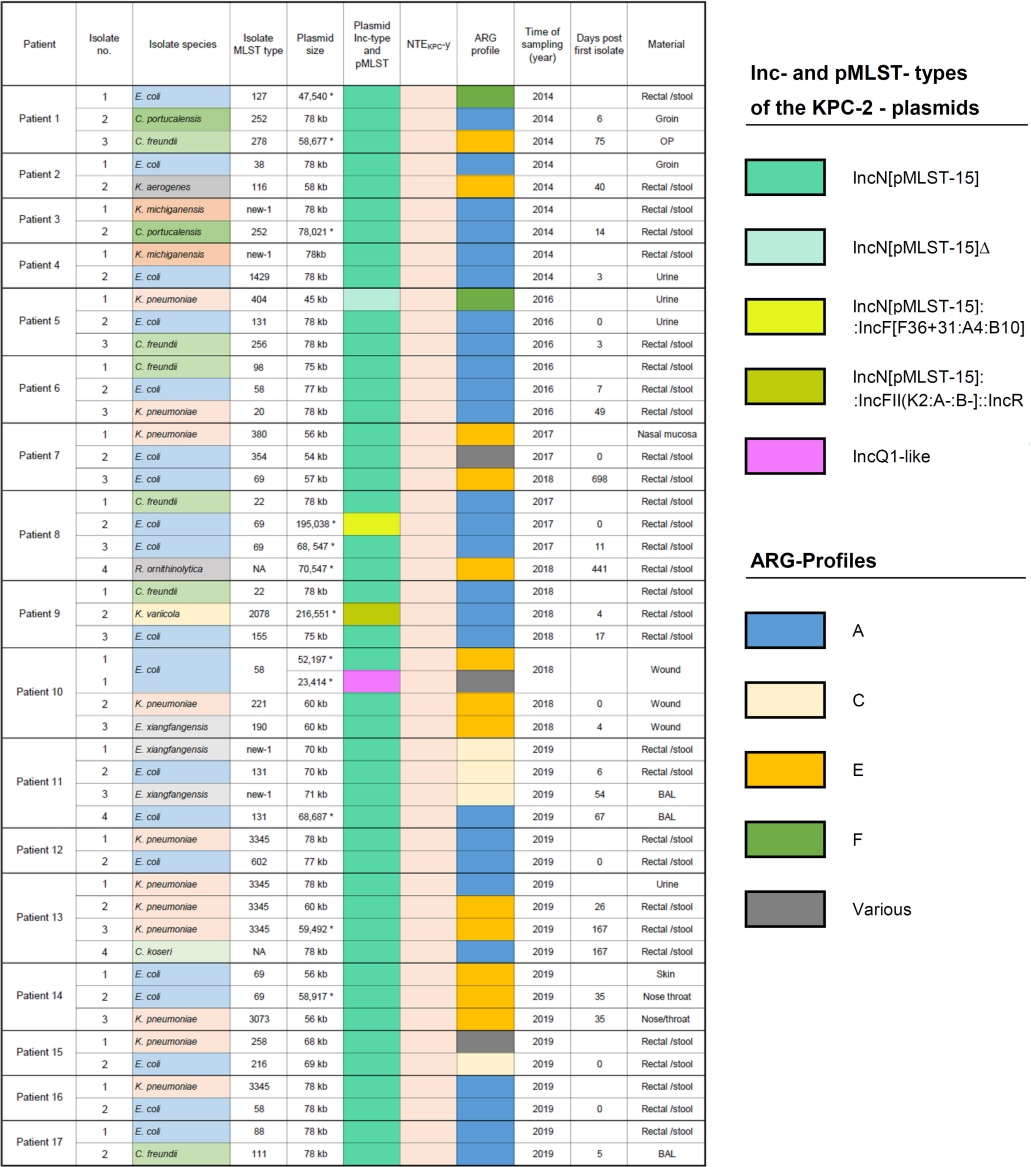
Patients with multiple KPC-2-producing species carrying the IncN[pMLST15] plasmid with the NTE_KPC_-Y element.

The region surrounding *bla*_KPC-2_ was unique and differed from both typical Tn*4401* structures (*tnpR*_Tn*4401*_-*tnpA*_Tn*4401*_-IS*kpn7–bla*_KPC-2_-IS*kpn6*) as well as from other known non-Tn*4401* elements (NTE*_KPC_*-I, II, and III) from previous studies (19, 25). We provisionally assigned it as NTE*_KPC_*-Y, to distinguish it from a newly described NTE*_KPC_*-X that is present on an IncN[pMLST15] plasmid in isolates from the United Kingdom (6). Fig. 2B illustrates the differences between the Tn*4401*-isoforms (a, b, and j) and the non-Tn*4401* elements, NTE*_KPC_*-Y and NTE*_KPC_*-X. In the Tn*4401*-isoforms, a Tn*4401*-resolvase, a Tn*4401*-transposase, and an IS*Kpn7*-transposase are present upstream of the *bla*_KPC-2_ gene, while for NTE*_KPC_*-Y, an IS*26* transposase, *aac (3)-IId*, three CDSs of unknown function, an IS*Cfr1,* and a Tn*2*-associated *bla*_TEM-1B_ gene are present. In NTE*_KPC_*-X, an IS*26* transposase*,* IS*Ecp1* element, and a Tn*2*-associated *bla*_TEM-1B_ gene are followed by an intact IS*Kpn6* downstream of the *bla*_KPC-2_ gene, whereby the IS*Kpn6* is split by an IS*5403* element. However, the combination of the *aac3-IId*, *bla*_TEM-1B,_ and *bla_KPC-2_* resistance genes on NTE*_KPC_*-Y is new and has not been hitherto detected. Fig. 3C shows the common insertion site of the *bla*_KPC-2_ with its surrounding elements and ARGs of the three KPC-2-bearing variants I, II, and III. The IncN[pMLST15] variant III plasmids in this study also harbored additional ARGs within its dynamic multi-drug resistance region (DMDR [Fig. 2C]). We designed specific PCR primer sets based on this unique genetic feature of the IncN[pMLST15]-plasmid and combined it with primer pairs for the pMLST15-backbone (Table S5). This composite PCR assay allowed unambiguous detection of this novel IncN[pMLST15] plasmid in diagnostic reference laboratories.

Several ARG profiles were clustered with respect to the 35-kb inserted segment (Regions IIA + IIB, Tables 2 and 3; Fig. 2). ARG-Profile A with 15 ARGs was the most common type (71%; 89/124) and found in 78% of the 77 KPC2-CPE-patients in 30 hospitals (Table S1). ARG-Profile E containing five ARGs was the next common type (14%; 17/124) and was found in 10 patients in seven hospitals. The remaining minor ARG profiles designated B, C, D, and F and others were detected in 18/124 isolates.

Plasmids carrying ARG profile A were significantly associated with outbreaks. They were predominant in the large multi-species outbreak in 2013/2014 and were recovered six times between 2016 and 2019, generally involving two–three patients colonized with different species at different hospitals, as well as in a single-species outbreak in 2019 (Table S4). Of note, all five IncN[pMLST15] plasmids in the rivulet water samples that were collected downstream of a sewage treatment plant had cluster profile A. In contrast, plasmids with ARG profile E were rarely associated with outbreaks but frequently occurred in multiple-species-colonization profiles of individual patients, indicating interspecies spread. The other profiles occurred mostly sporadically, suggesting that they were occasional minority variants resulting from deletions.

### Concurrent presence of IncN[pMLST15] plasmids in several species within individual patients or single samples

We observed that the NTE*_KPC_*-Y IncN[pMLST15] plasmid was present in multiple bacterial species in 17 individual patients, i.e., in 47 isolates from 10 different species (Fig. 3). Four patients were sampled during the 2013/14 outbreak, and the other 13 were obtained during the surveillance period. The number of the isolates per case was between two and four isolates comprising two to three species, often with a co-occurrence of *E. coli* and *K. pneumoniae*. The time span between the first and last isolate ranged between 4 and 167 days, and in two cases, it was even longer than a year (441 and 698 days). In four cases, different species with identical plasmids were detected from single samples such as rectal/stool swab (*n* = 3) and wound (*n* = 1). The IncN[pMLST15] plasmids within a patient frequently harbored identical ARG-profiles, mostly profile A.

### The IncN[pMLST15] plasmid harboring NTE_KPC_-Y is unique to Germany

To examine the global distribution of the *bla*_KPC-2_-bearing IncN[pMLST15] plasmid with the NTE_KPC_-Y-element, we compared it to sequences extracted from the NCBI Genbank and other publicly available databases. We subjected data for 476 wholly sequenced IncN plasmids from public databases (NCBI and PLSDB, 34,513 entries) to further analysis. Of these, 258/476 carry *repN_1* (true IncN plasmids) and are typable by pMLST. The most common IncN type was the pMLST7 (*n* = 64), followed by pMLST5, pMLST1, pMLST6, pMLST9, and pMLST15, with 58, 41, 40, 26, and 16 entries, respectively. We found 17 publicly available IncN[pMLST15] plasmids harboring a *bla*_KPC-2_. These originated mainly from clinical *K. pneumoniae* or *E. coli* isolates from Brazil, Israel, the United Kingdom, and France (Table S5). Comparative genomic analysis demonstrated that all plasmids carried *bla*_KPC-2/-3_ at a specific site inserted between a truncated *nuc* (endonuclease) gene and the *fipA* gene (Fig. 2D).

Surprisingly, all of the previously described IncN[pMLST15] plasmids are carriers of various *bla*_KPC-2_ elements, i.e., Tn*4401*-based or otherwise, regardless of their geographical origin (Table S6). We note that three similar plasmids with the novel NTE*_KPC_*-Y element were recently found in healthcare institutions in the Czech Republic that borders with Germany (accession no. CP070531, CP070538, and CP070545), suggesting regional dispersion.

## DISCUSSION

In this study, we detected the presence of *bla_KPC-2_* in different genera and species of *Enterobacterales* isolated from human and inanimate environments between 2013 and 2019 in Germany. This study analyzed isolates from 61 hospitals in the Federal State of Hesse serving a population of ~6.3 million inhabitants together with additional isolates from other regions in Germany including samples obtained from surface waters. We detected broad species-based dissemination of the *bla_KPC-2_* gene facilitated by intra- and inter-species horizontal gene transfer (HGT) of a single IncN[pMLST-15] plasmid. This transfer also led to the emergence of highly antibiotic-resistant bacteria that carry up to 14 other ARGs encoded on the same plasmid (Table 2). Previously, an association of KPC-2 with plasmids of different incompatibility (Inc) groups, such as FII, N, R, A/C2, P, L/M, Q1, FIB, U, FIA, X3, Q2, and N3, was reported, with FII and N being the most common (10). However, the proportion of the previously mentioned plasmids reported that are in fact cointegrates with an IncN plasmid, as detected in this study, is not known.

**Fig 4:**
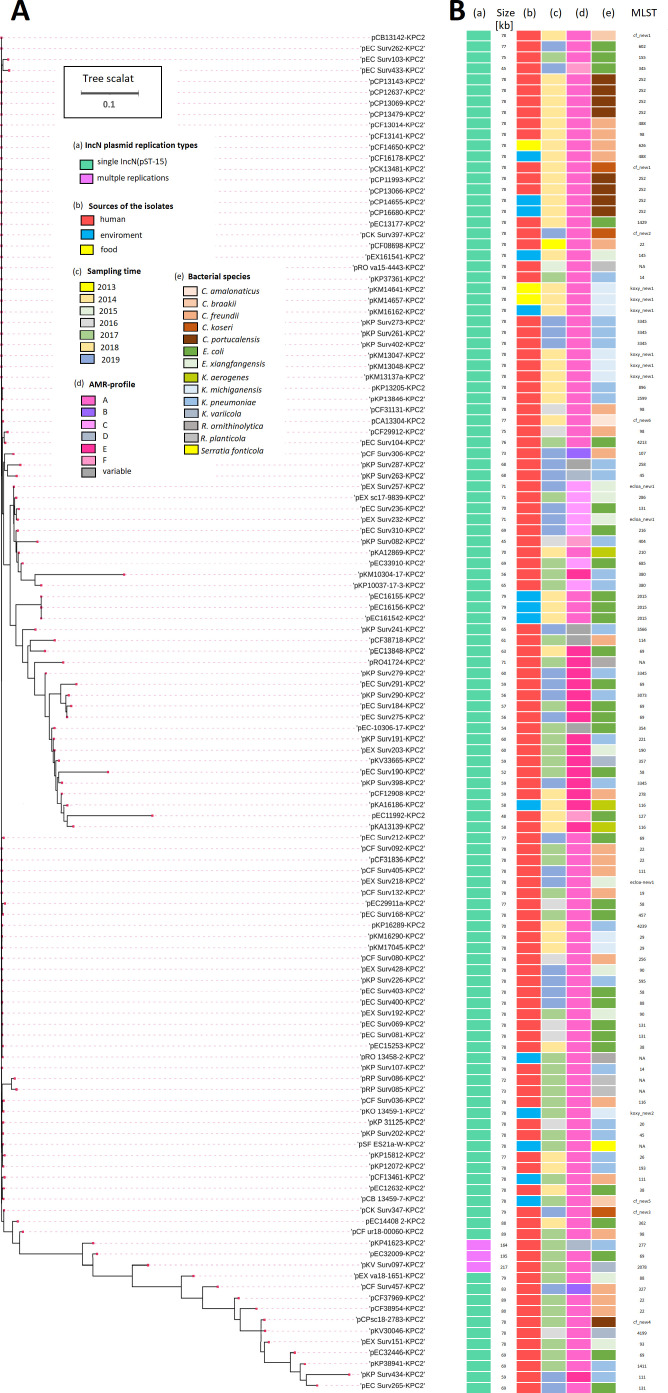
(**A**) Phylogenetic tree of the 124 IncN-KPC2-plamids either with a single IncN[pMLST-15] replication protein type (*n* = 121) or with a multiple replication protein type in co-integrated IncN[pMLST-15] (*n* = 3), which are IncN[pMLST-15]::IncF[F36-F31:A4:B10], IncN[pMLST-15]::IncF[K9:A -:B-], and IncN[pMLST-15]::IncF[K2:A-:B-]:IncR. Phylogenetic analysis showed that 84 of them were highly homologous, regardless of the time of isolation, source, and bacterial species. (**B**) Shown are the types, sizes, and AMR profiles of the IncN plasmids, as well as the sources of the isolates, the time of sampling, and the bacterial species.

Currently, 36% of all reported plasmids worldwide carry the *bla_KPC-2_* gene on the transposon element Tn*4401* (10), indicating the importance of transposons in transmission of this resistance. In our study, 94% were associated with NTE*_KPC_*-Y, a novel non-Tn*4401*-KPC2-element carried on IncN[pMLST15] plasmids, while only 6% of all isolates carry KPC-2 on a Tn*4401a* transposable element. The NTE*_KPC_*-Y element is unlikely to be mobile because it is always coupled to the presence of the IncN[MLST15] plasmid, that is, present even in cointegrates. Thus, the NTE*_KPC_*-Y element relies on the replication and maintenance properties of the broad host range IncN plasmid for effective dissemination and persistence in a range of different environments (Fig. 4).

In the 3-year SurvCARE surveillance study, IncN[pMLST15] comprised 88% of all KPC2-CPEs detected (Table 1). Plasmids carrying the ARG profile A significantly contributed to outbreaks, causing a large multi-species outbreak in southern Hesse in 2013/2014 (Table 1), six smaller multi-species outbreaks in several hospitals between 2016 and 2019, as well as a single-species outbreak in 2019 (Table S4). A largely identical NTE*_KPC_*-Y IncN[pMLST15] plasmid was previously reported from isolates obtained during outbreaks from hospitals in Berlin in 2016 (26) and more recently in Cologne (2020–2021) (Xanthopoulou K et al. ECCMID 2022, oral presentation O0234). Cumulatively, these data suggest that hitherto undetected transmission events may have resulted in the spread and persistence of this plasmid over large distances and time periods. The presence of the plasmid in environmental KPC2-CPE from surface water is deeply concerning as it emphasizes the presence of reservoirs in diverse ecological habitats. More recently, the detection of similar IncN[pMLST15] plasmids with NTE*_KPC_*-Y in *C. freundii* and *E. hormaechei* isolates from Ostrov, Koli, and Prague in the Czech Republic suggests multi-species regional, transboundary spread.

While the NTE*_KPC_*-Y element is clearly unique to Germany, similar NTE_KPC-2_ elements with unique compositions have been described in other countries such as Argentina, Brazil, China, Russia, and the United Kingdom (6, 17, 19). Their frequent association with broad host range IncN plasmids, which provide a versatile platform for the capture of these genes and its genetic plasticity, would facilitate transfer of large sets of ARGs even when antibiotics unrelated to carbapenems are used during therapy. An additional layer of complexity introduced by multi-species outbreaks exploits the ability of these plasmids to find appropriate bacterial hosts, e.g., *Citrobacter*, *Enterobacter*, and *Raoultella,* which are present in a wide variety of near-patient and community environments. This distribution is concerning as there are currently no effective strategies to force the elimination of these plasmids from their various hosts.

Our study has several limitations. The isolates submitted were part of mandatory reporting to regional healthcare authorities as well as voluntary contributions to the National Reference Center, and the study was limited in terms of access to clinical data of patients in the individual hospitals and healthcare institutions based on data protection considerations. The systematic surveillance study was geographically restricted to the state of Hesse and covers a catchment area of 21.115 km². In addition, environmental sampling at the individual institutions was not performed.

We provide evidence that the HGT properties of a single plasmid rather than clonal expansion of successful genetic lineages dominated the dissemination of *bla_KPC-2_* in Germany. Its location on a novel non-transposable Tn*4401* element (NTE*_KPC_*-Y) suggests that plasmid-encoded factors contribute to the endemicity and long-term persistence of KPC-2 in community-based, hospital, and environmental reservoirs. Data from studies that examine for the presence of plasmids of KPC2-CPE in community and hospital microbiomes, both in patients and the environment, are urgently needed to inform and complement current containment measures to enable the development of effective transmission-control protocols. These results emphasize the need, already now, to be aware of a prominent role of transmissible plasmids during CPE surveillance and their contribution to transmission events.

## MATERIALS AND METHODS

### Source of the KPC-2-carbapenemase-producing *Enterobacterales* isolates (KPC-CPE)

In the present study, we collected and sequenced 135 KPC-CPE isolates. Twenty-four isolates derived from 19 patients during a nosocomial outbreak in southern Hesse (2013–2014), twelve isolates from outbreak-associated hospital settings, 75 isolates from 59 patients during a Hesse-wide surveillance project (SurvCARE, 2017–2019), eight isolates from eight patients harboring a KPC-2-IncN-plasmid detected in the NRZ-collection (2014–2015), and five isolates from a screening project of carbapenem-resistant bacteria in surface water in Hesse 2017 (23).

### Sample size

To estimate the statistical power of our study, we used data on CPE prevalence from the National Reference Center for *Enterobacterales* at the Robert-Koch Institute in Germany between 2017 and 2019 (27 – 29). Based on these data, we expected to achieve a statistical power of 95% with a significance level (α) of 0.05 if we had a sample size of 145 isolates. Here, we performed genome sequencing of all 346 CPE samples obtained in this study, which increased the statistical power of our study to more than 99% with the same significance level.

### Phenotypic characterization of the bacterial isolates and plasmid conjugation assay

All bacterial isolates were identified using mass spectrometry MALDI-TOF MS (VITEK MS, Biomerieux, Nürtingen Germany), characterized for their phenotypical antimicrobial susceptibility using the automated VITEK2 system (Biomerieux, Nürtingen, Germany), and interpreted following EUCAST guidelines. For plasmid conjugation assays, the *E. coli* strain J53 was used as a recipient, as previously described (30).

### Whole-genome sequencing (WGS)

All isolates underwent WGS (Illumina MiSeq or NextSeq 500). A subset of the isolates was re-sequenced by long-read sequencing using the PacBio Single-Molecular-Real-Time (SMRT) or Nanopore technology to complete the genome. The Illumina library preparation and sequencing were carried out as previously described (31). Briefly, DNA sequencing libraries were prepared using the Nextera XT kit (Illumina MiSeq system (Illumina, Netherlands BV, Eindhoven, the Netherlands) according to the manufacturer’s introductions and sequenced either on a MiSeq instrument with 2 × 300 cycles or on a NextSeq instrument with 2 × 150 cycles. The SMRT-sequencing was carried out on a PacBio RSII machine (Pacific Biosciences, MenloPark, CA, USA), as described earlier (31). Sequencing using Nanopore technology with a MinION sequencer was performed as described previously (32).

### Genome assembly and plasmid-sequence completion

The Illumina-sequenced reads were assembled *de novo* using CLC Genomics Workbench version 8.0.1 (Qiagen, Aarhus A/S, Denmark) and/or SPAdes genome Assembler (33). For PacBio SMRT sequencing, the assembly was performed either using RS HGAP Assembly 3 or SMRT-Link Microbial Assembly v.10.1.0, using default parameters. The validity of each assembly was cross-checked using the RS_Bridgemapper.1 protocol, and each replicon was circularized independently. Finally, the circulated genome sequence was error-corrected by mapping of Illumina reads to the finished genomes using BWA (34), with subsequent variant calling using VarScan (35). A consensus concordance of QV60 was obtained for all of the genomes. For Nanopore sequencing, hybrid assembly was carried out using Unicycler v0.4.6.

For sequence finishing (completion) of the *bla*_KPC-2_-bearing plasmids of the isolates sequenced only by Illumina, contig-mapping and read-mapping against closed *bla*_KPC-2_-bearing plasmid-genomes from the SMRT-sequencing as references, by using SeqManPro (10.0), MAUVE (2.3.1), and CLC-Workbenches (8.0.1), were performed to complete the *bla*_KPC-2_-encoding plasmids of the remaining isolates that were only sequenced by short-read-sequencing.

### Genomic analyses

For genome-based species determination and MLST of the isolates, the in-house developed ASA³P pipeline was used (36). It was supplemented by using the websites PubMLST (https://pubmlst.org/databases/) and BIGSdb (https://bigsdb.pasteur.fr/cgi-bin/bigsdb/). Plasmid incompatibility groups (Inc), pMLST, antimicrobial resistance genes (ARG), and insertion sequences (IS) were identified using the Center for Genomic Epidemiology website (https://cge.cbs.dtu.dk/) (37, 38) and ISFinder (39). To annotate mobile antibiotic resistance genes and mobile elements, Galileo AMR of ARC Bio was used (40).

To determine the phylogenetic variation of the IncN plasmids, a maximum likelihood phylogeny was constructed using the Neighbor Joining Method based on the whole sequence alignment (CLC Genomics Workbench v.10.1.1) using default parameter settings. The phylogenetic tree was generated by MEGA6 (41).

Plasmid sequence comparison with the sequence of pCP13069-KPC2 as the reference was generated by using BLAST Ring Image Generator (BRIG) (42).

The NCBI Genbank database and other available complete sequence data were used to search the global distribution of the *bla*_KPC-2_-bearing IncN[pMLST15] plasmid with the specific non-Tn*4401*-KPC-element. IncN plasmid sequences (*n* = 476) and information from the plasmid database (PLSDB, https://ccb-microbe.cs.uni-saarland.de/plsdb/, as of 15 August 2022) were subjected to further analysis.

## Data Availability

All sequencing data have been deposited at NCBI GenBank under BioProject accession numbers PRJNA552260 and PRJNA692829.

## SUPPLEMENTAL MATERIAL

Tables S1 to S6; Fig. S1 spectrum.02564-23-s0001.xlsx Table S1 (Characteristics of KPC-2 type carbapenemase producers), Table S2 (Molecular characteristics of a novel non-Tn4401-Element [NTEKPC-Y]); Table S3 (Molecular characteristics of the KPC-2-producing Enterobacterales isolates with Tn4401 and other NTE elements); Table S4 (Distribution of the plasmids with ARG-Profile A); Table S5 (PCR-Primers used for stepwise detection of the IncN[pMLST15] plasmid); Table S6 (IncN[pMLST15] and other related plasmids carry the replicase allele repN_7 with an insertion hot spot between their fipA and nuc [truncated] genes); Fig. S1 (Diversity of blaKPC-2-positive plasmids and conjugation studies).

ASM does not own the copyrights to Supplemental Material that may be linked to, or accessed through, an article. The authors have granted ASM a non-exclusive, world-wide license to publish the Supplemental Material files. Please contact the corresponding author directly for reuse.
